# Probiotic Supplements on Oncology Patients’ Treatment-Related Side Effects: A Systematic Review of Randomized Controlled Trials

**DOI:** 10.3390/ijerph18084265

**Published:** 2021-04-17

**Authors:** Miguel Rodriguez-Arrastia, Adrian Martinez-Ortigosa, Lola Rueda-Ruzafa, Ana Folch Ayora, Carmen Ropero-Padilla

**Affiliations:** 1 Faculty of Health Sciences, Pre-Department of Nursing, Jaume I University, Av. Sos Baynat, 12071 Castellon de la Plana, Spain; arrastia@uji.es (M.R.-A.); afolch@uji.es (A.F.A.); ropero@uji.es (C.R.-P.); 2 Research Group CYS, Faculty of Health Sciences, Jaume I University, Av. Sos Baynat, 12071 Castello de la Plana, Spain; 3 Emergency Department, Miguel Servet University Hospital, Puerto de Isabel la Catolica, 50009 Zaragoza, Spain; adriaanortigosa@gmail.com; 4 Department of Functional Biology and Health Sciences, Faculty of Biology-CINBIO, Campus Lagoas-Marcosende, University of Vigo, 36310 Vigo, Spain

**Keywords:** drug therapy, gut microbiota, neoplasms, probiotics, radiotherapy, systematic review

## Abstract

Cancer affects more than 19.3 million people and has become the second leading cause of death worldwide. Chemo- and radiotherapy, the most common procedures in these patients, often produce unpleasant treatment-related side effects that have a direct impact on the quality of life of these patients. However, innovative therapeutic strategies such as probiotics are being implemented to manage these complications. Thus, this study aimed to evaluate the efficacy of probiotics supplements as a therapeutic strategy in adult oncology treatment-related side effects. A systematic review of randomized controlled trials was conducted in PubMed, Scielo, ProQuest and OVID databases up to and including January 2021, following the PRISMA guidelines. The quality of the included studies was assessed by the Jadad Scale. Twenty clinical trials published between 1988 and 2020 were included in this review. Seventeen studies (85%) revealed predominantly positive results when using probiotics to reduce the incidence of treatment-related side effects in oncology patients, while three studies (15%) reported no impact in their findings. This study sheds some light on the significance of chemotherapy and radiotherapy in altering the composition of gut microbiota, where probiotic strains may play an important role in preventing or mitigating treatment-related side effects.

## 1. Introduction

According to the World Health Organization (WHO), cancer is the second leading cause of death, affecting more than 19.3 million people and claiming 10 million lives worldwide, and the number of new cases is expected to double by 2040 [1]. This disease is diagnosed differently in men and women, with one in every five people developing cancer at some point in their lives, resulting in the death of one in every eight men and one in every eleven women diagnosed with cancer. In this sense, breast, colorectal, lung, cervical, and thyroid cancer are the most common cancers in women, while lung and prostate cancer are the most common in men [2].

There are diverse therapeutic strategies to reduce cell proliferation and disease progression, with surgery, chemotherapy, radiotherapy, and, more recently, immunotherapy and hormone therapy being the most commonly used treatments [3,4]. These treatments have significant side effects, particularly chemotherapy and radiotherapy, which is why it is frequently necessary to use combination treatments to increase effectiveness, despite the fact that this strategy multiplies side effects [5]. As a result, cancer treatments have the greatest impact on cells with the highest rate of cell division, resulting in low cell counts in blood cells, which manifests as anemia, infections, and bleedings. Likewise, gastrointestinal cells are also altered, resulting in nausea, vomiting, diarrhea, taste disturbances, mucositis, and swallowing difficulties [6,7], which cause many patients to postpone or discontinue their treatments [8].

Chemo- and radiotherapy modify the composition of intestinal microbiota in a process known as dysbiosis, which is often associated with biochemistry and immunologic disorders in the gastrointestinal tract [9,10]. Multiple strategies are being developed to modify microbiota with the underlying idea of propelling this dysbiosis toward eubiosis or the hemostasis of the gut microbiota in order to prevent or inhibit cancer progression [11,12]. In this regard, it has been reported that paclitaxel, a mitosis inhibitor, is able to increase matrix metalloproteinase 9 (MMP9) and tumor necrosis factor-alpha (TNF-α) levels and alter bacterial diversity in female mice colon [13]. Probiotics, defined as live microorganisms that provide a health benefit to the host when administered in adequate amounts [14], have been shown to be effective in the management of diarrhea and constipation, as well as highly effective in the treatment of inflammatory bowel diseases by improving bowel function [15,16]. For example, a probiotic mixture improved altered intestinal tight junction levels in mice with dextran sodium sulfate (DSS)-induced colitis [17]. Consequently, probiotics containing one or more strains could indeed restore the composition of altered gut microbiota and improve certain parameters, leading to significant homeostasis in animal models of obesity, Parkinson’s disease, and depression [15,18,19]. Similarly, immune function may improve after the administration of a probiotic combination. Treatment with Bifidobacterium longum, Lactobacillus lactis, and Enterococcus faecium significantly reduced the occurrence of radio- chemotherapy-induced oral mucositis, as well as increased CD4+, CD8+, and CD3+ T cells in oncological patients [20]. In that manner, 5-fluorouracil-induced intestinal mucositis has also shown an improvement after probiotic treatment by reducing TNF-α, IL-6, and IFN-γ levels in mice [21].

In this context, corticosteroids and antiemetics are key elements in oncology to be used prior to the administration of chemotherapy to avoid side effects [5]. However, relatively little is understood about including probiotics in this preventive regimen due to beneficial results in intestinal disorders and altered immunity, which could be of great interest in reducing certain oncology treatment-related side effects, such as diarrhea, mucositis, or constipation. Therefore, this review aims to evaluate the efficacy of probiotic supplements to ameliorate chemo- and radiotherapy-related side effects in adults.

## 2. Materials and Methods

### 2.1. Design

A systematic review of randomized controlled trials was undertaken in January 2021, following the Preferred Reporting Items for Systematic Reviews and Meta-Analyses (PRISMA) guidelines (Supplementary File 1) [22]. This review used a structured Patient–Intervention–Outcome (PIO) question as follows [23]: “In adult oncology patients (P), what is the efficacy of probiotics supplements (I) on treatment-related side effects (O)?” The protocol for this review was not registered.

### 2.2. Search Strategy

The electronic databases included PubMed, Scielo, ProQuest, and OVID, using natural and structured language in the following search strategy: (((((probiotics [Title/Abstract] OR probiotics [MeSH Terms]) OR lactobacillus [Title/Abstract]) OR bifidobacterium [Title/Abstract]) OR lactobacillus [MeSH Terms]) OR bifidobacterium [MeSH Terms]) AND ((((radiotherapy [Title/Abstract] OR chemotherapy [Title/Abstract]) OR chemotherapy [MeSH Terms]) OR radiotherapy [MeSH Terms]) OR radiation [Title/Abstract]). This search strategy was adapted for use across databases (Appendix A). “Randomized clinical trial”, “humans”, and “adult:19+ years” search filters were applied for this search strategy.

### 2.3. Selection Criteria

The following inclusion criteria were used: (i) randomized clinical trials, (ii) published in English or Spanish, (iii) related to the aim of the study; the use of probiotics supplements on adult oncology-related treatments and side effects; and (iv) published until January 2021. Likewise, the exclusion criteria included (i) studies on other pathologies than cancer or symptoms related to cancer treatments, (ii) symbiotics and other treatment combinations, (iii) re-publications, and (iv) studies with animals. No articles were excluded after quality appraisal.

### 2.4. Data Screening

Initially, the two authors (MR, AM) performed a first screening by titles and abstracts, following the selection criteria independently and in duplicate. Once a third author (CR) double-checked the screening and discussed any discrepancy, a full-text reading was performed for their quality appraisal by authors.

### 2.5. Quality Appraisal

The quality of selected articles was assessed by two researchers independently (MR, AM). Any disagreements on quality ratings were discussed with a third author (CR) and a consensus was reached. The Jadad Scale of Clinical Trials was used to assess the methodological quality of experimental human studies included. This is a scale with five simple items and it has known reliability and external validity. A score below 3 points indicate low quality based on (i) the quality of randomization, (ii) double blinding, and (iii) drop-outs extracted from each study [24].

### 2.6. Data Abstraction and Synthesis

Consecutively, the relevant data from the included studies were extracted and tabulated according to (i) authors, (ii) country, (iii) population, (iv) probiotic strains, (v) variables, (vi) measures, and (vii) main findings.

## 3. Results

### 3.1. Characteristics of Selected Studies

**Figure 1:**
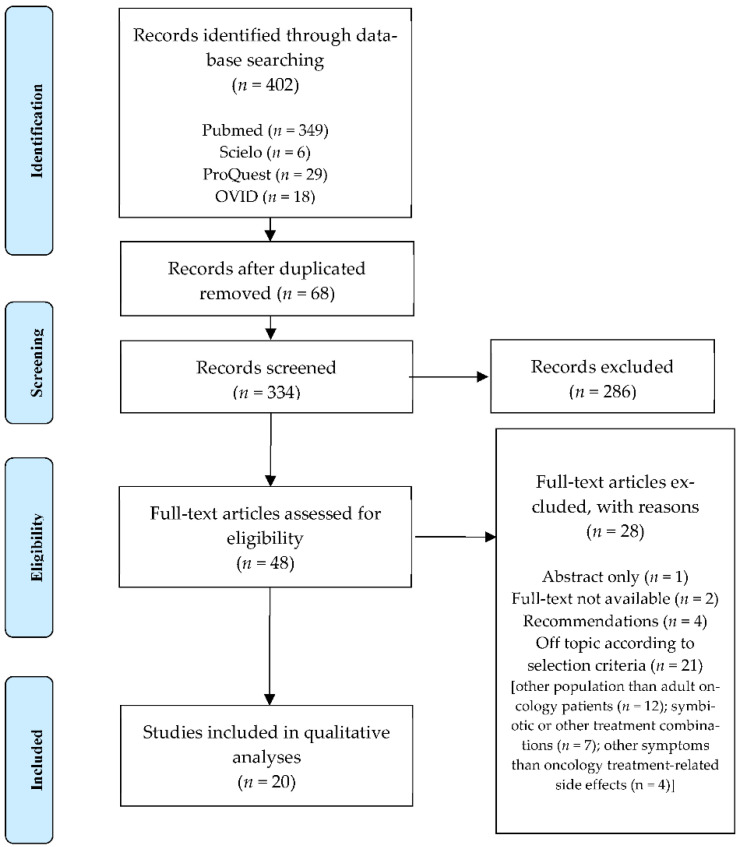
Flowchart depicting the article selection process.

Firstly, a total of 402 articles were retrieved through databases searching (PubMed (*n* = 349), Scielo (*n* = 6), ProQuest (*n* = 29) and OVID (*n* = 18)), from which 68 papers were discarded by duplicity. After title, abstract, and full-text screening, a total of 314 articles were excluded following the selection criteria. Twenty studies were included in this review (Figure 1).

All trials and patients’ characteristics are summarized in Table 1. On the whole, all individuals were treated using conventional cancer therapy methods, such as radiotherapy (*n* = 11, 55%), chemotherapy (*n* = 6, 30%), or both (*n* = 3, 15%). In some studies, sex was not specified (*n* = 2, 10%), and only women were included in studies dealing with specific carcinomas of the female reproductive tract (*n* = 4, 20%), such as endometrial, vaginal, uterine, and cervical cancers. The age range of the patients ranged from 18 to 75 years old (with a mean age of 57.41 years), enrolling a total of 2508 participants. All studies included were published between 1988 and 2020, and 15 studies (75%) were not registered in any clinical trial registry. Most of these studies were conducted in Asia (*n* = 9), Europe (*n* = 8), but also in America (*n* = 2) and Oceania (*n* = 1). As regards the use of probiotics, 10 of the selected studies (50%) used a single probiotic strain, while the remaining 10 (50%) used two or more probiotics combined. The presentation and forms of administration varied from study to study, with the most commonly used forms being capsules, gelatine, and yoghurt. The time of administration as well as the dose administered to the patients were also varied, which ranged from 1 to 24 weeks and 10^6^ to 10^11^ CFU/day, respectively. Finally, 17 studies (85%) revealed predominantly positive results when using probiotics to reduce the incidence of treatment-related side effects in oncology patients, while three studies (15%) reported no impact in their findings.

The data synthesis revealed four categories related to the use of probiotic supplements for treatment-related side effects in clinical oncology. For that matter, these categories would study the effects of probiotic treatments for different treatment-related side effects in oncology such as gastrointestinal side effects, immune-related side effects, inflammatory side effects, and performance status-related side effects. These categories are described below.

### 3.2. Gastrointestinal Side Effects

Probiotics have been shown to be effective in the treatment of some common oncology treatment-related gastrointestinal adverse reactions, as demonstrated in 11 of 20 trials (55%) [25,26,27,28,29,30,31,33,34,35,36]. The main adverse effects identified and treated were mainly diarrhea, with other drawbacks being abdominal pain, nausea and vomiting, constipation, bloating, abdominal distension, and lactose intolerance caused by chemotherapy. The most commonly probiotic strains used along these studies were *Lactobacillus acidophilus*; *L. rhamnosus* GG ATCC 53103; and *L. casei* var. *rhamnosus*. Likewise, other probiotic strains used in combination were (*L. acidophilus* LA-5 along with *Bifidobacterium animalis* subsp. *lactis* BB-12), (*L. acidophilus* BMC12130, *L. casei* BCMC12313, *L. lactis* BCMC12451, *B. bifidum* BCMC02290, *B. longum* BCMC02120 and *B. infantis* BCMC02129), (*B. infantis*, *L. acidophilus*, *Enterococcus faecalis* and *Bacillus cereus*), (*L. acidophilus* LAC-361 and *B. longum* BB-536), (*L. acidophilus* plus *B. bifidum*), and (*L. casei*, *L. plantarum*, *L. acidophilus*, and *L. delbruekii* subsp. *thermophilus*; *B. longum*, *B. breve*, and *B. infantis*; *Streptococcus salivarius* subsp. *thermophilus*). The duration of treatment ranged from 1 to 24 weeks.

Conversely, 2 trials (10%) showed inconclusive results for their benefits to control stool constituency and flatulence, although their findings were promising to prevent radiotherapy-induced diarrhoea [32,37]. The probiotic strains used in these studies included: *L. acidophilus* NCDO1748, and (*S. thermophilus*, *L. delbrueckii* subsp. *bulgaricus*, and *L. casei* DN-114 001). The treatment for these studies ranged from 1 to 6 weeks and were observed only in women.

### 3.3. Immune-Related Side Effects

Despite having only one study [38], positive results of the probiotics on immune-related side effects have also been observed. A combination of probiotic strains (*Bifidobacterium*, *Lactobacillus* and *S. thermophilus*) was used for 1 to 2 weeks, in which patients improved their immune and nutritional status as well as rehabilitation, showing improved cellular immune parameters and tolerance to abdominal pain, bloating and diarrhoea. These authors used glutamine along with fish oil in their treatment as it has been shown to enhance epithelial cell growth and repair of intestinal mucous membrane, prevent bacterial translocation and reduce barrier injury, among others, which may actually be able to work synergistically with probiotics to protect the intestinal mucosa barrier and reduce permeability. In this manner, radiation-induced injuries may be alleviated by these probiotic strains, while other eco-nutrients feed the intestinal membrane.

### 3.4. Inflammatory-Related Side Effects

Impact on inflammatory-related side effects such as oral mucositis was also reported in three trials (15%). Among these studies, two trials [20,40] showed positive and effective results in reducing the severity of oral mucositis when using different probiotic strains: (*B. longum*, *L. lactis,* and *E. faecium*) and *L. brevis* CD2. The treatment period for these studies was from 1 to 7 weeks. However, De Sanctis et al. (2019) [39] did not notice any significant changes in the severity of oral mucositis with *L. brevis* CD2, although their treatment lasted only 1 week due to premature closure of patient accrual. While it is true that radio-chemotherapy-induced mucositis is a complex process and further prospective studies are needed to explore oral microbiota modulation in reducing its incidence, the findings of Jiang and collaborators (2019) [20] and Sharma and collaborators (2012) [40] strongly underpinned the probiotics used as a plausible strategy to manage mucositis-associated pain and reduce its incidence.

### 3.5. Performance Status-Related Side Effects

Concerning to the impact of probiotics in patients’ general well-being and activities of daily life, three trials (15%) evaluated their effects over a 4-week treatment period [41,42,43]. Two of these studies used a single probiotic strain, *S. salivarius* M18 and *L. casei* LC9018 respectively, and the remaining study used a combination of *L. acidophilus*, *L. rhamnosus*, *B. longum,* and *Saccharomycesboulardii*. In line with the findings of Shao and collaborators (2014) [38], not only did Doppalapudi and collaborators (2020) [42] and Vesty and collaborators (2020) [41] observe clinical improvements driven by probiotic-induced changes in the oral microbiota but also a potential mechanism to improve these performance status-related side effects throughout other modulating host immune response and microbial interactions. Having said that, only one study [43] assessed the effect of probiotics in malignant pleural effusion, which is one of the most common complications in lung cancer. This complication can have a severe impact on patient performance and shortened survival, but interestingly, *L. casei* LC9018 has been shown to be a useful adjuvant in the treatment of this type of cancer and to prevent this complication.

### 3.6. Quality Assessment

**Figure 2:**
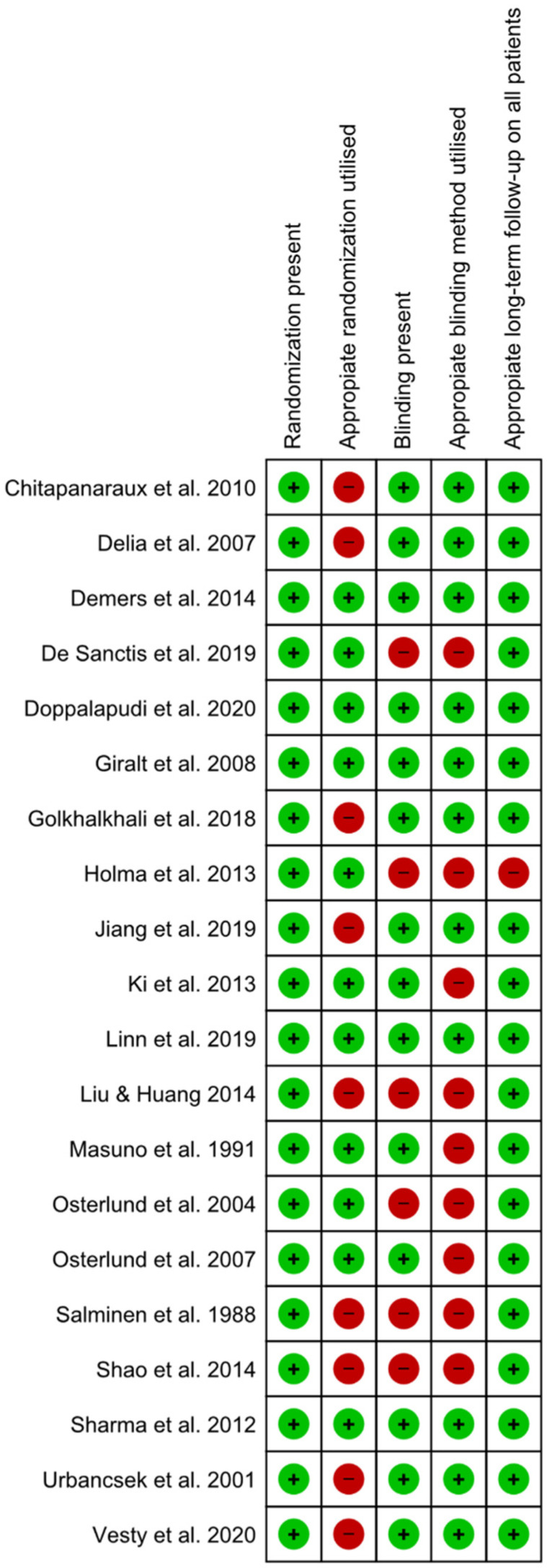
Risk of bias summary using the Jadad Scale for each included study.

On the Jadad Scale, the average quality of the analyzed studies was 3.75 (Figure 2). Its reporting quality varied from 2 (in four studies), 3 (in two studies), 4 (in nine studies), and 5 (in five studies), with none of them having an inappropriate reporting quality (lower than 1). At last, four of the studies reviewed received support from different manufacturers, indicating the possibility of a sponsorship bias [26,31,36,40].

## 4. Discussion

This review was aimed to evaluate the efficacy of probiotics supplements as a therapeutic strategy for treatment-related side effects in adult oncology patients. After analyzing 20 randomized controlled trials, our findings showed the beneficial effects that probiotic may have in a range of common treatment-related side effects, which have a direct impact of the oncology patients’ quality of life. In this manner, 11 of 20 studies (55%) observed positive outcomes among gastrointestinal adverse effects management such as diarrhea, abdominal pain, nausea, and vomiting among others. Similarly, another six studies (30%) reported promising results in the control of immune and inflammatory responses, as well as other side effects related to their overall well-being and daily life activities. These findings further support the idea of previous reviews [44,45], suggesting that microbiota plays a key role in the pathogenesis of some treatment-related side effects, although further evidence is needed to determine their safety and accuracy [46,47].

The studies included in this review were heterogenous in the use of probiotic strains, where *Lactobacillus acidophilus* (LA-5, BMC12130, LAC-361, and NCDO1748) was the most widely used strain among other 15 different strains, both in single strain [29,37] and multiple strain trials [25,26,27,28,31,34,42]. This heterogeneity added to the number of cancers included may explain some of the between-studies variability of the results [47]. Another possible explanation may be the interindividual diversity of the microbiota composition, where personalized medicine might well contribute to predicting the most suitable probiotic strain for the individual [48]. In this vein, strong evidence suggests that the efficacy of probiotics is strain-specific as well as disease-specific, and therefore, these factors should be considered when recommending the best probiotic for the patient [49]. Furthermore, the duration of treatment may also have to be considered to demonstrate probiotic clinical position in the oncology of treatment-related side effects, 4 weeks being the most common duration of treatment among the studies included. These results are consistent with the findings of De Sanctis and collaborators (2019) [39], who stated that a probiotic treatment period of less than 4 weeks may not be sufficient to observe and confirm their beneficial effects. However, to date, there are not standardized procedures available on the minimum treatment duration for the selected probiotic strain in order to observe positive outcomes, as it requires time to promote gut microbiota re-shaping and, as a result, the beneficial effect [50].

In reference to the treatment-related side effects, authors such as Delia and collaborators (2007) [34], Golkhalkhali and collaborators (2018) [26], as well as Osterlund and collaborators (2007) [33] among others, concur that the use of probiotics and microbial cell preparations improves the intestinal immune barrier, particularly intestinal IgA responses. In line with the results of other studies, these probiotic strains are able to stabilize the intestinal microbial environment and improve the permeability of the intestinal barrier, leading to a reduction in inflammatory response and promoting changes in the intestinal flora [51,52]. This promotes an ideal environment for the growth of non-pathogenic bacteria, helping to protect epithelial cells, the process of apoptosis, and some cytoprotective processes [53]. Interestingly, similar results were found using probiotic strains such as *Lactobacillus*, *Bifidobacterium,* or *Streptococcus* along with other eco-nutrients such as glutamine and fish oil [38]. These results match those observed in recent preclinical studies [54,55], where the colonization of this bacteria genera enhanced the immune and anti-inflammatory response to radiation, forming an enteric–intestinal barrier that increased the thickness of the intestinal flora. Moreover, the optimization of the medium promotes the life of living microorganisms, which can restore the balance of a radiation-damaged microecosystem by repairing the intestinal membrane, inhibiting the growth of intestinal pathogens, and reducing endotoxin production [56]. These probiotic strains are antioxidant agents that act by eliminating free radicals produced by ionization and preventing lipid oxidation, thereby prioritizing the repair and regeneration of the cell membrane, DNA, and proteins, resulting in their high efficacy in reducing abdominal pain, flatulence, and diarrhea, as these authors highlight in their findings [38,56].

In accordance with these findings, Holma and collaborators (2013) [30] underline the importance of fecal pH and methane production in this type of patient, where intestinal microbiota plays a central role in the incidence of unpleasant side effects such as diarrhea and constipation, bloating, or abdominal inflammation. These results confirm the association between the higher production of elements such as methane and microbiota, where a higher production of methane is associated with a lower incidence of diarrhea and a methane deficiency is associated with a higher incidence of abdominal discomfort [57]. In this context, the results showed that the *L. rhamnosus* GG ATCC 53,103 strain did not alter the production of pH or methane, as opposed to studies such as Salminen and collaborators (1988) [37], in which *L. acidophilus* NCDO1748 was administered and increased flatulence was observed, pointing directly to the lactulose content as a non-absorbable substrate, a mechanism favoring the production of methane and probiotic absorption. In this sense, Osterlund and collaborators (2004) [35] provide information on lactose intolerance caused by low intestinal villus height in relation to its depth of treatment, resulting in malabsorption syndrome and therefore hindering the production of diarrhea, flatulence, and abdominal pain [58]. In line with the overall evidence, it is worth noting how adverse effects can be managed by modifying gut microbiota and methane production mechanisms [30,59]. Replacing lactulose with another non-absorbable substrate would not cause diarrhea and would, in turn, allow the amount of methane to be controlled to achieve balance in intestinal transit, vary the amount of substrate administered, and greatly improve or even reduce the number of treatment doses administered to patients [35,57].

On the other hand, oral mucositis and oral health stand as one of the most treated side effects as they significantly reduce the patients’ quality of life [60]. In that matter, probiotics such as *B. longum* (BCMC02120, BB-536), *L. lactis* BCMC12451, *E. faecium*, and *L. brevis* CD2 have shown to reduce the incidence of severe oral mucositis by promoting the growth and protection of the bacterial flora and, as a result, decreasing the number of adverse effects, severity, and incidence of mucositis [20,40,41,42]. These findings are in agreement with those of Vesty and collaborators (2020) [41], who identified that using *S. salivarius* M18 improved patients’ quality of life by reducing the number of oral infections (candidiasis) and adverse effects (mucositis, diarrhea) that these patients experienced after their treatments. However, recent research found that the effects of *L. brevis* CD2 were unable to confirm its beneficial impact for severe oral mucositis, though one possible explanation for these findings could be the premature closure of patient accrual [39]. Lastly, it is also interesting to note the effect of probiotics on other side effects of these patients such as pleural effusion, which can severely affect their performance status and even shorten their life expectancy. Only Masuno and collaborators (1991) [43] evaluated the use of the *L. casei* LC9018 strain against this complication, demonstrating promising results in controlling pleural effusions by reducing the number of malignant cells at the pleural level, which are supported by preclinical models [61,62].

### Limitations

That being said, there are some limitations to bear in mind when interpreting the findings of this study. Fifteen of the analyzed studies were not registered, and therefore there may be a risk of reporting bias, whereas these studies are consistent trials on the Jadad Scale. On the other hand, heterogeneity in strains, length of treatment, and population could be confounding factors, and hence, generalizations should be made with caution. As a result of this heterogeneity in strains, interventions, and data collection methods, neither meta-analysis nor meta-regression were considered in this review. Given the small number of studies included, further work is still needed on the clinical position of probiotic supplements in adult oncology treatment-related side effects, in particular to determine the efficacy of individual probiotic strains, which could help to compare strains and lead more closely to preventive approaches.

As a whole, this review contributes to the existing literature, providing evidence of the current clinical position of probiotics supplements for some common treatment-related side effects in adult oncology patients. Despite the main findings of these studies concluded in terms of the safety and efficacy of probiotics supplements for the treatment or prevention of these side effects, further research with larger groups, specific strains, and duration of treatment is needed to conclude the beneficial effects for each of these side effects. More broadly, research is needed to determine the effects of individual and combined probiotic strains in order to draw confident conclusions about their benefits for both general oncology treatment-related side effects and specific cancers. Future research will be particularly interesting in determining how the use of probiotics and prebiotics may enhance the beneficial effect of the first to improve therapeutic responses in patients with cancer.

## 5. Conclusions

This study has shown that some probiotic strains (*L. acidophilus*, *L. casei*, *B. longum,* or *L. rhamnosus* among others) are a valid therapeutic strategy in some common treatment-related side effects in adult oncology patients, using both single or multiple strain combinations for at least 4 weeks of treatment. The beneficial variation between the different strains in the selected studies has been similar, which is why all of them represent a possible strategy for complications such as gastrointestinal side effects, immune or inflammatory side effects, and performance status-related side effects. Furthermore, despite its exploratory nature, this study provides some insight into the importance of chemotherapy and radiotherapy, inducing major changes in the composition of microbiota, where these probiotic strains may play an important role to prevent or treat such complications.

### Implications for Clinical Practice

Common treatment-related side effects such as diarrhea, vomiting, mucositis, or abdominal pain are unpleasant for patients who have to undergo chemo- or radiotherapy treatments. Although more research is clearly needed, it has been shown that the gut microbiota plays a key role in immunity, and therefore, probiotics could be considered as a potential therapeutic strategy for treating and preventing these complications in immunocompromised cancer patients. Certain probiotic strains (e.g., *Lactobacillus* or *Bifidobacterium*) have shown to be safe and effective for some of these effects secondary to chemo- and radiotherapy, but also to significantly enhance immune response in these patients. Rather than concluding on this topic, this review provides a common ground to explore more in detail the use of certain probiotic strains for common side effects such as pleural effusions, which have a profound impact on the quality of life and life expectancy of these patients.

## Figures and Tables

**Table 1:** Overview of clinical selected articles.

Reference	Country (TN)	Population (*n*)	Probiotic Strains	Dose and Treatment Period	Variables	Measures	Main Findings
	Gastrointestinal side effects						
Linn et al. (2019) [25]	Myanmar (TCTR20170314001)	54 cervical cancer patients	Single: *L. acidophilus* LA-5 plus *B. animalis* subsp. *lactis* BB-12	1.75 × 10^9^ CFU/day 3 weeks	The incidence of RID, abdominal pain and use of anti-diarrheal	RID severity was assessed by the common terminology criteria for adverse events, and the severity of abdominal pain was assessed by the CTCAE	The incidence and severity grades of RID was significantly reduced, as well as abdominal pain and the use of anti-diarrheal drug
Golkhalkhali et al. (2018) [26]	Malaysia (IRCT201106156814N1)	140 colorectal cancer patients	Multi: *L. acidophilus* BMC12130, *L. casei* BCMC12313, *L. lactis* BCMC12451, *B. bifidum* BCMC02290, *B. longum* BCMC02120 and *B. infantis* BCMC02129	3 × 10^10^ CFU/day 8 weeks	Effect of supplementation in QOL, chemotherapy side effects, and inflammatory markers in colorectal cancer	QOL was assessed by EORTC QLQ-C30 scale. CRP measure was used for the evaluation of inflammatory markers	Patients’ QOL was improved, reducing certain inflammatory biomarkers and relieving diarrhea, nausea, and vomiting
Liu and Huang (2014) [27]	China	100 cancer patients	Multi: *B. infantis*, *L. acidophilus*, *E. faecalis* and *Bacillus cereus*	0.5 × 10^6^ CFU/day 4 weeks	Efficacy, side effects, and difference between the two groups	Wexner Score was used to measure changes	Functional constipation during chemotherapy was effectively and safely treated
Demers et al. (2014) [28]	Canada	246 pelvic cancer patients	Multi: *L. acidophilus* LAC-361 and *B. longum* BB-536	1 × 10^10^ CFU/day 15 weeks	Severity of RID, intestinal pain, and the usage of anti-diarrheal medication	Diarrhea severity was evaluated by WHO grading scale and the abdominal pain according to NCI scale. Stool consistency was measured by Bristol scale	RID was reduced at the end of the treatment. Nutritional assessment appears to reduce global digestive symptomatology
Ki et al. (2013) [29]	USA	40 prostate cancer patients	Single: *L. acidophilus*	1 × 10^8^ CFU/day 3 weeks	Rectal volume and volume change of the rectum	CT, MVCT, and PVCR for checking the percentage volume change of the rectum	*L. acidophilus* was effective for reducing excessive gas and exacerbated bloating or distension
Holma et al. (2013) [30]	Finland	143 colorectal cancer patients	Single: *L. rhamnosus* GG ATCC 53103	1 × 10^9^ CFU/day 24 weeks	Gastrointestinal symptoms during chemotherapy, methane production and fecal pH	Both fecal and breath samples were analyzed to assess methane production and its pH. Gastrointestinal symptoms and OLT were used to assess chemotherapy injuries	*L. rhamnosus* GG reduced diarrhea during chemo and did not affect significantly to methane production
Chitapanarux et al. (2010) [31]	China	63 cervical cancer patients	Multi: *L. acidophilus* plus *B. bifidum*	2 × 10^9^ CFU/day 6 weeks	Incidence and severity of diarrhea, stool characteristics, and the use of anti-diarrheal medication	Stool consistency was analyzed in laboratory and hematological toxicities were measured by CTC	Incidence of RID and the usage of anti-diarrheal medication were reduced, while the stool consistency was improved
Giralt et al. (2008) [32]	Spain	85 endometrial adenocarcinoma patients	Multi: *S. thermophilus*, *L. delbrueckii* subsp. *bulgaricus*, and *L. casei* DN-114 001	1 × 10^8^ CFU/day 6 weeks	Severity of RID, and inflammatory intestinal conditions	Diarrhea was measured by CTC. The fecal calprotectin was analyzed in laboratory, using an enzyme-linked immunoassay	The oral supplementation may result in a modest clinical benefit for stool consistency
Osterlund et al. (2007) [33]	Finland	150 colorectal cancer patients	Single: *L. rhamnosus* GG ATCC 53103	1–2 × 10^10^ CFU/day 24 weeks	Chemotherapy dose intensity and tolerability	A diary kept by the patients and by a physician was used to assess side effects	The frequency of severe 5-FU-based chemotherapy-related diarrhea was reduced
Delia et al. (2007) [34]	Italy	490 sigmoid, rectal, or cervical cancer patients	Multi: *L. casei*, *L. plantarum*, *L. acidophilus*, and *L. delbruekii* subsp. *thermophilus*; *B. longum*, *B. breve*, and *B. infantis*; *S. salivarius* subsp. *thermophilus*	4.5 × 10^11^ CFU/day 4 weeks	Clinical symptoms after radiation therapy, concomitant medications, and AEs. Incidence and severity of RID	Daily bowel movements were monitored, and the severity of gastrointestinal toxicity was measured as WHO grading	This treatment constitutes a safe option to protect patients against RID even in the setting of intestinal inflammation
Osterlund et al. (2004) [35]	Finland	150 colorectal cancer patients	Single: *L. rhamnosus* GG ATCC 53103	1–2 × 10^10^ CFU/day 24 weeks	Lactose intolerance, effect of probiotic, treatment-related toxicity, and nutritional status	OLT, symptom questionnaire, and Subjective Global Assessment of Nutritional Status questionnaire	*L. rhamnosus* GG had an impact on lactulose intolerance symptoms, but not in the frequency of hypolactasia
Urbancsek et al. (2001) [36]	Austria	205 cancer patients	Single: L. casei var. rhamnosus	1.5 × 10^9^ CFU/day 1 week	Efficacy for treatment of diarrhea	Bowel movements, diarrhea grading, feces ratings by the investigator, and patient diarrhea ratings	Probiotic therapy produced a highly favorable benefit/risk ratio in RID
Salminen et al. (1988) [37]	Finland	24 cervix or uterus carcinoma patients	Single: *L. acidophilus* NCDO1748	2 × 10^9^ CFU/day 6 weeks	Frequency and severity of intestinal side effects, the usage of anti-diarrheal medication	Data on diarrhea, abdominal pain, meteorism, flatulence, vomiting, defecation frequency and usage of anti-diarrheal medication was collected	*L. acidophilus* NCDO1748 appears to prevent RID, but increases the incidence of flatulence due to its substrate
	Immune-related side effects						
Shao et al. (2014) [38]	China	46 ARE patients	Multi: *B. lactobacillus* and *S. thermophilus*	0.5 × 10^9^ CFU/day 2 weeks	Nutritional status, abdominal pain, flatulence, and diarrhea	Level of serum albumin, prealbumin occurrence rate of abdominal pain, flatulence, diarrhea, and blood PCT in fast blood was measured	Patients’ immune status was improved, and the tolerance of enteral nutrition could be better for the bowel function and the patients’ rehabilitation
	Inflammatory-related side effects						
De Sanctis et al. (2019) [39]	Italy (NCT01707641)	75 HNC patients	Single: *L. brevis* CD2	2 × 10^9^ CFU/day 1 week	Incidence of severe oral mucositis and of requirement for enteral nutrition	Incidence and severity of treatment-related dysphagia; patient QOL; body weight loss during; the incidence and time-course of treatment-related pain	The effects of *L. brevis* CD2 were not able to confirm the beneficial in reducing OM in patients with HNC
Jiang et al. (2019) [20]	China	99 NC patients	Multi: *B. longum*, *L. lactis* and *E. faecium*	1 × 10^7^ CFU/day 7 weeks	Patients’ immunity status, composition and abundance of bacterial communities	Total bacterial genomic DNA extraction and high-throughput sequencing and efficacy at the end of treatment	Immune response was significantly increased and severity of OM was reduced
Sharma et al. (2012) [40]	India	200 HNC patients	Single: *L. brevis* CD2	2 × 10^9^ CFU/day 4 weeks	Incidence and severity of OM and chemo-radiotherapy-related adverse effects	FACT-HN questionnaire was used for QOL. Saliva samples were collected for pro-inflammatory biomarkers	*L. brevis* CD2 proved to be safe and efficacious in reducing the incidence of severe OM
	Performance status-related side effects						
Vesty et al. (2020) [41]	New Zealand (ACTRN12617000710325)	17 HNC patients	Single: *S. salivarius* M18	3.5 × 10^9^ CFU/day 4 weeks	Bacterial community networks and oral probiotic viability	Sample collection, oral health assessments, probiotic viability, DNA extraction and sequencing preparation, bioinformatic analyses, and network analyses were used	Oral probiotics to modulate host immune responses and microbial interactions is a promising mechanism to improve oral health
Doppalapudi et al. (2020) [42]	India (CTRI/2018/02/011812)	86 HNC patients	Multi: *L. acidophilus*, *L. rhamnosus*, *B. longum* and *Saccharomycesboulardii*	1.5 × 10^9^ CFU/day 4 weeks	Difference in salivary count pre- and post- intervention, and prevalence at the end of treatment	Saliva samples were collected for isolation, count, and identification of *Candida*	The probiotic bacteria were effective in reducing oral *Candida* spp.
Masuno et al. (1991) [43]	USA	95 lung cancer patients	Single: *L. casei* LC9018	0.2 × 10^7^ CFU/day 4 weeks	Median survival, common side effects, and changes in the severity of each symptom	CT scan and pleural fluid cytologic specimens were examined. Laboratory tests were performed	LC9018 appears to be a useful agent for the treatment of lung cancer and prevent pleural effusions

TTN: Trial number; CFU: Colony-forming unit; L.: Lactobacillus; B.: Bifidobacterium; E.: Enterococcus; S.: Streptococcus; RID: Radiation-induced diarrhea; CTCAE: Common Terminology Criteria for Adverse Events; QOL: Quality of life; EORTC: European Organization for Research and Treatment of Cancer; CRP: C-reactive protein; NCI: National Cancer Institute; CT: Computed tomographic; MVCT: Megavoltage computed tomography; PVCR: Percentage volume change of the rectum; OLT: Oral lactulose tolerance; CTC: Common Toxicity Criteria; AE: Adverse effects; ARE: Acute radiation enteritis; PCT: Procalcitonin; HNC: Head and neck cancer; NC: Nasopharyngeal carcinoma; OM: Oropharyngeal mucositis; FACT-HN: Functional Assessment of Cancer Therapy Head and Neck.

## Data Availability

The dataset used and/or analyzed in this study is available from the corresponding author on reasonable request.

## Supplementary Materials

The following are available online at https://www.mdpi.com/article/10.3390/ijerph18084265/s1, Supplementary File 1.

## Appendix A

**Table A1:** Search strategies for each database used.

	Pubmed	Scielo	Proquest	Ovid
Probiotics	(((((probiotics [Title/Abstract] OR probiotics [MeSH Terms]) OR lactobacillus [Title/Abstract]) OR bifidobacterium [Title/Abstract]) OR lactobacillus [MeSH Terms]) OR bifidobacterium [MeSH Terms])	((ti:probiotics OR ab:probiotics OR kw:probiotics) OR (ti:lactobacillus OR ab:lactobacillus OR kw:probiotics) OR (ti:bifidobacterium OR ab:bifidobacterium OR kw:bifidobacterium))	((((AB,TI(probiotics) OR MESH(probiotics)) OR AB,TI(lactobacillus)) OR AB,TI(bifidobacterium)) OR MESH(lactobacillus) OR MESH(bifidobacterium))	((probiotics OR lactobacillus) OR bifidobacterium)
Oncology treatments	AND ((((radiotherapy [Title/Abstract] OR chemotherapy [Title/Abstract]) OR chemotherapy [MeSH Terms]) OR radiotherapy [MeSH Terms]) OR radiation [Title/Abstract])	AND (((ti:chemotherapy OR ab:chemotherapy OR kw:chemotherapy) OR (ti:radiotherapy OR ab:radiotherapy OR kw:radiotherapy)) OR (ti:radiation OR ab:radiation OR kw:radiation))	AND (((AB,TI(chemotherapy) OR AB,TI(radiotherapy)) OR MESH(chemotherapy)) OR MESH(radiotherapy))	((chemotherapy OR radiotherapy) OR radiation)
